# Prevalence of erectile dysfunction and associated factors among newly diagnosed ART naïve men living with HIV: a cross sectional study in Mwanza, Northwestern Tanzania

**DOI:** 10.3389/fruro.2025.1657553

**Published:** 2025-09-16

**Authors:** Shabani Iddi, Dismas Matovelo, Karol J. Marwa, Benson R. Kidenya, Haruna Dika, Samuel E. Kalluvya

**Affiliations:** ^1^ Department of Physiology, Weill Bugando School of Medicine, Catholic University of Health and Allied Sciences, Mwanza, Tanzania; ^2^ Department of Obstetrics and Gynecology, Weill Bugando School of Medicine, Catholic University of Health and Allied Sciences, Mwanza, Tanzania; ^3^ Department of Pharmacology, Weill Bugando School of Medicine, Catholic University of Health and Allied Sciences, Mwanza, Tanzania; ^4^ Department of Biochemistry and Molecular Biology, Weill Bugando School of Medicine, Catholic University of Health and Allied Sciences, Mwanza, Tanzania; ^5^ Department of Internal Medicine, Weill Bugando School of Medicine, Catholic University of Health and Allied Sciences, Mwanza, Tanzania

**Keywords:** HIV, antiretroviral naïve males, erectile dysfunction, associated factors, Tanzania

## Abstract

**Background:**

Erectile dysfunction (ED) is a frequent finding in men living with human immunodeficiency virus (HIV) (MLWH) and this remains a major concern because of its negative impact on the quality of life of those affected. There is limited data about the magnitude of ED and associated factors among MLWH in Tanzania. Thus this study was aimed to determine the prevalence of ED and associated factors among newly diagnosed antiretroviral therapy (ART)-naive MLWH in Mwanza, Northwestern Tanzania.

**Methods:**

A cross-sectional study was conducted among 373 newly diagnosed ART-naïve MLWH attending voluntary counseling and testing centers of four selected hospitals in Mwanza region who were consecutively enrolled and subjected to thorough clinical and general physical examination, including anthropometric measurements. A pre-structured questionnaire was used to collect socio-demographic characteristics and clinical data. ED was assessed using the International Index of Erectile Function–5. Serum total testosterone, follicle-stimulating hormone, luteinizing hormone and estradiol were estimated. Data were entered in Microsoft Excel, cleaned and analyzed using STATA version 15.

**Results:**

Of the 373 analyzed participants with a median age of 40 [IQR: 33–46] years, ED was found in 56.3% (95% CI 51.2%–61.3%), whereas the majority presented with mild (45.2%) to mild-moderate (40.0%) ED. The median testosterone was significantly lower in men with ED as compared with men without (294.5 [135–469] versus 482 [191–602] ng/ml; p*<*0.001). In a multivariate logistic regression analysis, ED showed significant association with World Health Organization (WHO) clinical stage 4 for HIV infection (AOR 3.2; 95% CI 1.1–9.2; p=0.032), low testosterone level (AOR 1.9; 95% CI 1.2–3.0; p=0.010), and being non-self-employed (AOR 3.7; 95% CI 2.0–7.0; p<0.001).

**Conclusion:**

ED was found in more than half of ART naïve MLWH. The majority had a mild to mild-moderate ED. There was a significant association between ED and WHO clinical stage 4 for HIV infection, low testosterone level, and being non-self-employed. This finding emphasizes the need to routinely screen for early detection and management of ED in care and treatment center (CTC) clinics.

## Introduction

Erectile dysfunction (ED) is defined as the inability to achieve or maintain a satisfactory penile erection during sexual intercourse (1, 2). The main causes of ED are categorized as either psychogenic (depression, stress and anxiety) or organic (hormonal, neurological, vascular and tissue problems) (3). Hypogonadism is one of the cause of ED, so ED can be caused by any category of hypogonadism including that which occur due to aging (4, 5), non-infectious conditions (obesity, diabetes, hypertension, cancers, malnutrition among others) (4, 6–9), anemia (4, 7), common acute and chronic illnesses (10), weight loss (11), invasion of the glands by pathogens (Hepatitis virus, Cytomegalovirus, HIV among others) (2, 12), cigarette smoking, using drugs, such as opiates, megestrol acetate, methadone (4, 13, 14) and steroids (15), hyperprolactinemia as well as primary and secondary hypogonadism (12, 16, 17). Some of the herbal medicines are known to possess antifertility properties through various mechanisms including inhibiting 5-alpha reductase, a factor that converts testosterone into dihydrotestosterone, reducing gonadotropins and testosterone secretion, increasing the testosterone affinity for sex specific proteins among others (18). Since traditional herbal medicine and complementary alternative medicine are commonly used by treatment naïve HIV patients (19, 20), they can therefore be one of the cofactors for hypogonadism.

The prevalence of ED is higher in men living with human immunodeficiency virus (HIV) (MLWH) than in the general population reaching to 74% in some studies (21). The pathogenesis of ED in these patient is not clear. Hypogonadism is one of the most frequent endocrine disorders in HIV infected men. Low levels of testosterone leads to reduced sexual desire and ED, however some studies have reported poor relationship of testosterone to these sexual function parameters (22, 23). Further, antiretroviral therapy (ART) had been implicated as the cause of sexual problems including erectile dysfunction (24–26) but with contradicting results in other studies (21, 27, 28). In addition, psychological (depression) or neurological (infection, dementia) problems often cause ED (3, 29–33).

Even though ED is more prevalent in MLWH than in the general population, little consideration has been given worldwide to the diagnosis and management of ED among MLWH, and Tanzania inclusive. There is paucity of evidence on the magnitude and factors associated with ED in MLWH in Tanzania. This study is aimed to determine the prevalence of ED and its associated factors among newly diagnosed MLWH, in Mwanza, Northwestern Tanzania.

## Materials and methods

### Study design, setting and period

A hospital-based, multi-center, cross-sectional study involving newly diagnosed HIV positive males was conducted at Voluntary Counseling and Testing (VCT) centers at Sekou-Toure Regional Referral Hospital (STRRH), Bugando Medical Centre (BMC), Nyamagana District Hospital (NDH) and Magu District Hospital (MDH), Mwanza, Tanzania from January 2020 and August 2022.

### Study population and eligibility criteria

All newly diagnosed HIV positive (diagnosed as per WHO guidelines 2015) males aged 18 years and above who attended the VCTs during the study period were included in the study. Patients with previous history of gonadal dysfunction, taking drugs known to affect hormone levels (i.e. androgens, sex steroids, dehydroepiandrosterone, antiandrogens, anabolic agents, GnRH agonists and psycholeptic agents), having chronic liver disease, coinfection with hepatitis B virus (HBV) and hepatitis C virus (HCV) chronic kidney injury, tuberculosis and Diabetes mellitus (DM) that could serve as confounders were excluded from this study.

### Sample size and sampling procedure

Sample size for this study was calculated using Kish-Leslie formula (1965) (34)


$$n=\frac{Z\sigma^{2}x(p)\times (1-p)}{\epsilon^{2}}$$

Where:

n = minimum sample size
Zσ = Z score level of significance (1.96)
P = Prevalence from previous study
ϵ = Precision of the study (set at 5% or 0.05)

Using the prevalence of ED of 37.8% from a study done in Nigeria (35) gave the minimum sample size of 361 participants. A total of 388 participants were enrolled in this study in order to take care of non-response. However, 373 participants were included in the final analysis. Fifteen (3.9%) participants were excluded as they had not completed the IIEF-5 questionnaire for erectile dysfunction. A convenient sampling technique was used to enroll study participants, where subjects were recruited as they were coming at each hospital until the sample size for the study was attained. At the completion of data collection the distribution of participants was: STRRH 105 (28.1%), BMC 110 (29.5%), NDH 132 (35.4%), and MDH 26 (7.0%).

### Data collection and laboratory procedure

All participants were evaluated clinically by comprehensive history taking and a general physical examination including anthropometric measurements such as waist circumference (WC) and body mass index (BMI). Socio-demographic data including age, employment status, marital status and herbal medicine use status (whether they used any herbal medicine within the past six months or not) were collected using a pre-structured questionnaire. Height was measured in the upright standing position using a calibrated stadiometer. Body weight was measured with minimal clothing by using a standard calibrated weighing scale, and BMI was then calculated by the formula: weight in kilograms divided by height in meters squared. WC was measured at the approximate midpoint between the lower margin of the last palpable rib and the top of the iliac crest using flexible plastic tape and was calculated as an average of 3 measurements. Anthropometric parameters were measured by a single trained research assistant at each hospital.

ED was studied by using the validated Swahili translated 5-item version of the International Index of Erectile Function (IIEF-5) questionnaire (36–40). The items in the IIEF-5 questionnaire focus on erectile function and intercourse satisfaction (36, 41). All responses to the IIEF-5 questions were rated on a 5-point scale, with a score of 1 representing the worst and 5 the best response. ED was classified into five severity levels as follows: 1-7: Severe ED, 8-11: Moderate ED, 12-16: Mild-moderate ED, 17-21: Mild ED and 22-25: No ED.

Five milliliters (mls) of venous blood sample was collected from each of the study participants between 8.00 AM and 11.00 AM and serum was harvested. The serum was used for the estimation of total testosterone (TT) hormone, follicle stimulating hormone (FSH), luteinizing hormone (LH) and estradiol levels. The serum samples were stored at -20°C for not more than 30 days until analyzed.

The hormonal tests were done using chemiluminescence immunoassay (CLIA) techniques. The CLIA kits were sourced from the Snibe Co., Ltd, Shnzhen, China. Serum hormones (TT, FSH, LH and estradiol) were estimated using the fully-automated chemiluminescence immunoassay analyzer model Maglumi 2000 (Snibe Diagnostic, China) following CLIA principles and the kit manufacturer’s instructions. The CD4+ count was assessed by flow cytometry (Roche diagnostics).

Hypogonadism was defined as a serum TT level of<300ng/dl or a serum TT level of ≥300 ng/dl with high FSH (>12 mlU/L) or LH (>12 mlU/L) level (42). Eugonadism was defined as normal TT and normal FSH and LH levels. Compensatory hypogonadism was defined as normal TT but high FSH or LH levels. Primary hypogonadism was defined as low TT levels with high FSH and LH, while secondary hypogonadism was defined as low TT with low or normal FSH or LH (42, 43).

### Data analysis

Data were cleaned and checked for completeness and consistency and then corrected. The data were coded and entered into Microsoft Excel and then transported to STATA software, version 15 (Texas, USA) for analysis. For descriptive statistics results were expressed in tables, bar graphs and pie charts. Categorical variables were summarized using frequencies and percentages while continuous variables were summarized using median with interquartile range (IQR). Graphical distribution plots and Shapiro-Wilk test was used to assess the normality of data distribution. The associations between categorical variables and ED were determined using a Pearson’s Chi-square test or Fisher’s exact test where appropriate. To assess the significance of difference when comparing various patient characteristics (Age, BMI, Waist circumference, CD4 cell count and TT) between participants with ED and those without ED we used Wilcoxon rank-sum test. Univariate followed by multivariate logistic regression models were used to determine risk factors for ED whereby factors with a p value less than 0.2 in the univariate were subjected to multivariate logistic regression analysis. In all analyses, the statistical significance was set at a p-value of less than 0.05.

### Ethical considerations

Ethical approval was granted by the Joint Catholic University of Health and Allied Sciences and Bugando Medical Center (CUHAS/BMC) research ethics and review committee with ethical clearance certificate number CREC/407/2019. Permission to carry out the study was also sought from the appropriate hospital authorities. The study participants provided written informed consent before participating in the study. No information was shared with any third party, and numbers were used in the questionnaires instead of personal identifiers. The copies of the hormone assay results report were shared to the clinicians, and all participants diagnosed with hypogonadism and ED were advised to report to the clinicians for advice and further management.

## Results

A total of 388 participants were enrolled in this study, however, the final analysis included 373 newly diagnosed ART naïve MLWH with a median age [IQR] of 40 [33–46] years. Fifteen participants were excluded from the final analysis as they did not complete the IIEF-5 questionnaire for ED assessment. Of the 373 participants, 35% (132/373) were from NDH, 29% (110/373), 28% (105/373) and 7.0% (26/373) from BMC, STRR and MDH respectively. The majority (58.7%, 219/373) of participants were in the age range 31–45 years, were married (64.3%, 240/373) and reported to have not used herbal medicine in the past six months (66.5%, 248/373). The median BMI and WC of the study participants were 21.1 [19.4–23.5] kg/m^2^ and 80 [76–83] cm respectively. About three-quarter of participants had BMI between 18.5 and 24.9 (normal), more than fifty percent had a WC of less than 81 cm (lower) and three-quarters, 75.1% (280/373) were self-employed (**Table 1**).

**Table 1:** Socio-demographic characteristics of study participants (N = 373).

Variable	Frequency (n)/Median	Percent (%)/IQR
Median age (Years)	40	[33 – 46]
Age groups (Years)		
18-30	52	13.9
31-45	219	58.7
≥ 46	102	27.4
Median BMI (kg/m^2^)	21.1	[19.4 – 23.5]
BMI category (kg/m^2^)		
<18.5	55	14.9
18.5 – 24.9	262	70.8
25-29.9	49	13.2
≥ 30	4	1.1
Median Waist Circumference (cm)	80	[76 – 83]
Waist circumference category (cm)		
<81	206	55.2
81 – 93	156	41.8
94 – 115	11	3.0
Marital status		
Married	240	64.3
Single	81	21.7
Divorced/separated	52	14.0
Herbal medicine use		
Yes	125	33.5
No	248	66.5
Employment status		
Non self employed	68	18.2
Self Employed	280	75.1
Unemployed	25	6.7

### Clinical characteristics of study participants

Among 267 study participants who had CD4+ data, the median CD4+ count was 297 [164.0–409]. The majority, 62.5% (233/376) of the study participants were grouped in stage one of the disease according to World Health Organization HIV staging. The median testosterone was 376 [165–553] and the majority (56.3%, 210/373) had testosterone levels above normal. Hypogonadism was present in nearly half (48.8%, 182/373) of study participants (**Table 2**).

**Table 2:** Clinical characteristics of study participants (N = 373).

Variable	Frequency (n)/Median	Percent (%)/IQR
Median CD4 count (cells/µl)	297	[164 – 409]
CD4 category		
>350	103	38.6
<200	80	30.0
200-350	84	31.5
WHO clinical stage		
Stage 1	233	62.5
Stage 2	58	15.5
Stage 3	60	16.1
Stage 4	22	5.9
Median Total Testosterone (TT) (ng/ml)	376	[165 – 553]
TT category		
≥300	210	56.3
<300	163	43.7
Hypogonadism		
Yes	182	48.8
No	191	51.2

### Prevalence of erectile dysfunction

**Figure 1:**
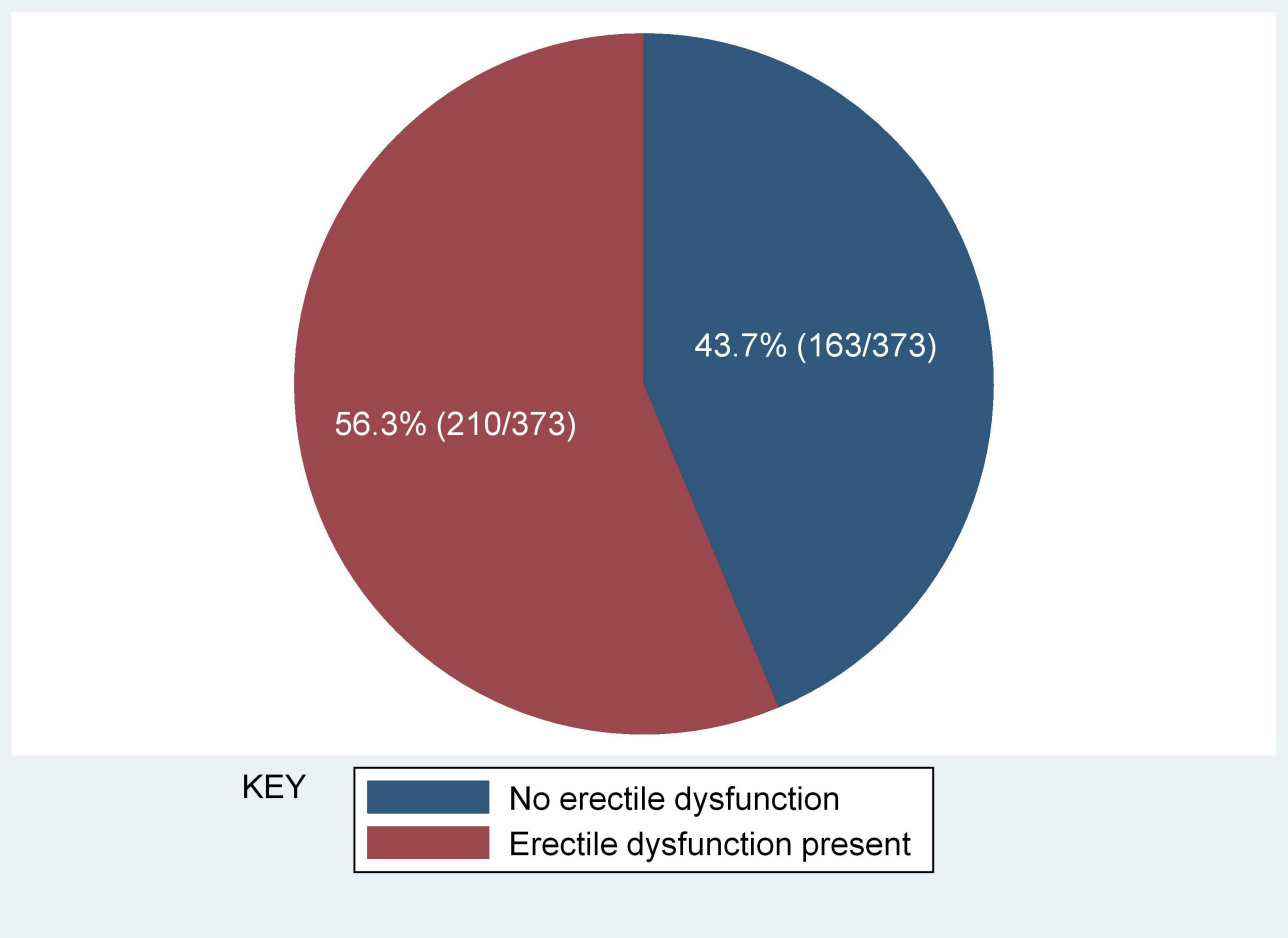
Prevalence of erectile dysfunction among study participants.

**Figure 2:**
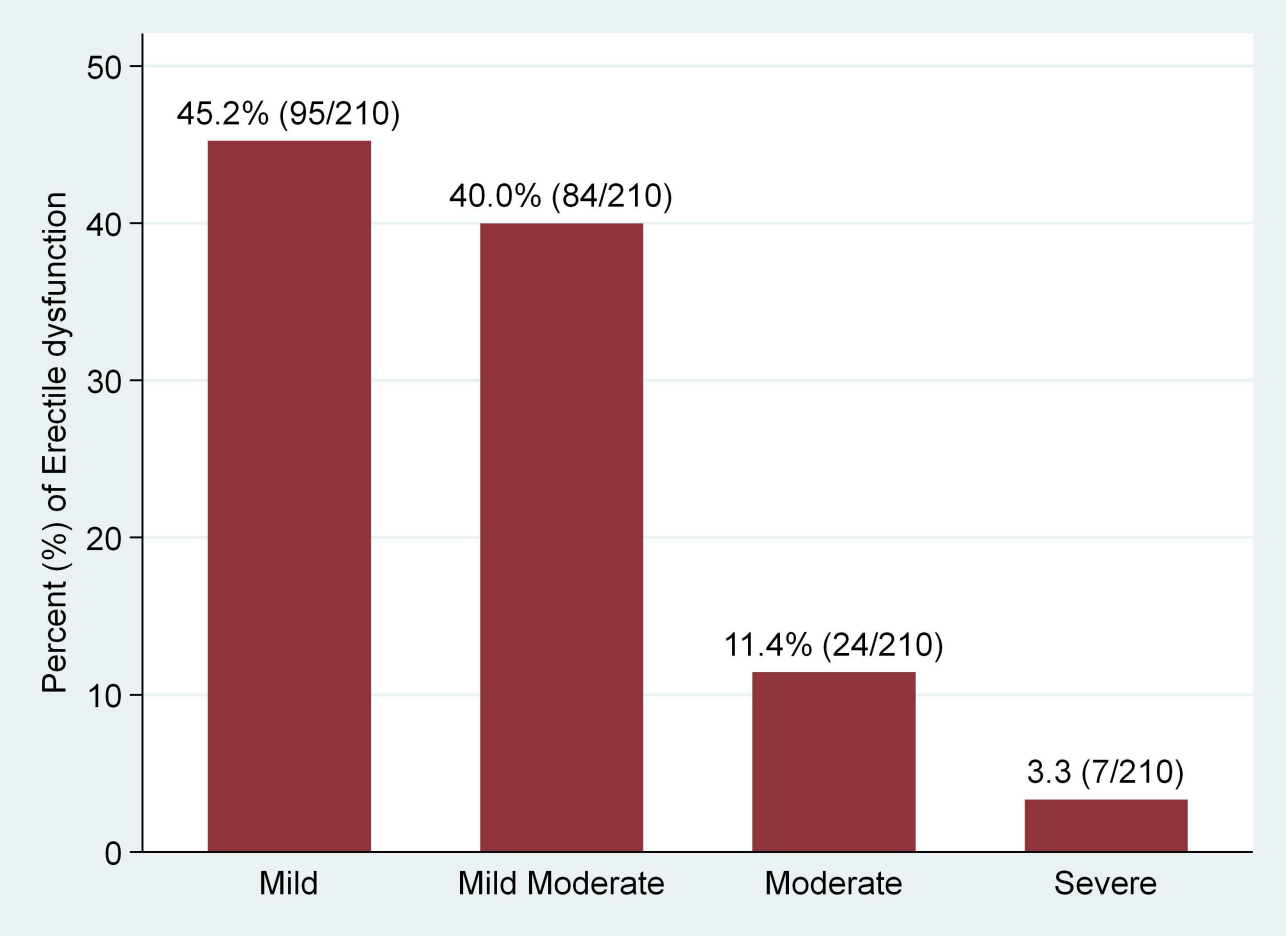
Categories of ED among participants with ED.

Erectile dysfunction (ED) was found in 56.3% (*95%* CI 52.1%–61.3%)) of the study participants (**Figure 1**). Severe ED was observed in 7/210 (3.3%), moderate in 24/210 (11.4%), mild-moderate 84/210 (40.0%), and mild in 95/210 (45.2%) of the participants with ED, respectively (**Figure 2**). The median [IQR] of age was significantly higher among men with ED (41.0 [35–48] years) as compared with men without (38.0 [32-45] years) (p = 0.009) (Two-sample Wilcoxon rank-sum (Mann-Whitney) while the median [IQR] of serum TT level was significantly lower among men with ED as compared to men without (294.5 [135–469] versus 482 [191–602] ng/ml; p*<* 0.001*)* (Two-sample Wilcoxon rank-sum (Mann-Whitney) test). The median BMI, Waist circumference and CD4 count did not differ significantly between the two groups (**Table 3**).

**Table 3:** Socio-demographic and clinical characteristics of study participants according to the erectile function status (N = 373).

Characteristics	With erectile dysfunction [N = 210 (56.3%)] n (%)	Without Erectile dysfunction [N = 163 (43.7%)] n (%)	p-Value
Median age (Years) (Median [IQR])	41 [35–48]	38[32–45]	0.009†
Age groups (Years)			
18-30	26 (50.0)	26 (50.0)	0.117
31-45	118 (53.9)	101 (46.1)	
≥ 46	66 (64.7)	36 (35.3)	
Median BMI (kg/m^2^) (Median [IQR])	20.9 [9.3–23.4]	21.4 [19.5–23.8]	0.224†
BMI category (kg/m^2^)			
<18.5	34 (61.8)	21 (38.2)	0.439*
18.5–24.9	148 (56.5)	114 (43.5)	
25-29.9	25 (51.0)	24 (49.0)	
≥ 30	1 (25.0)	3 (75.0)	
Median Waist Circumference (cm)	80 [76–84]	80 [77–83]	0.699†
Waist circumference category (cm)			
<81	113 (54.8)	93 (45.2)	0.782*
81–93	90 (57.7)	66 (42.3)	
94–115	7 (63.6)	4 (36.4)	
Marital status			
Married	146 (60.8)	94 (39.2)	0.056
Single	40 (49.4)	41 (50.6)	
Divorced/separated	24 (46.2)	28 (53.8)	
Herbal medicine use			
Yes	79 (63.2)	46 (36.8)	0.056
No	131 (52.8)	117 (47.2)	
Employment status			
Non self employed	51 (75.0)	17 (25.0)	0.002
Self Employed	144 (51.4)	136 (48.6)	
Unemployed	15 (60.0)	10 (40.0)	
CD4 count (cells/µl)	289 [148–404]	309 [209–421]	0.245†
CD4 category			
>350	57 (55.3)	46 (44.7)	0.297
<200	53 (66.3)	27 (33.7)	
200-350	48 (57.1)	36 (42.9)	
WHO clinical stage			
Stage 1	118 (50.6)	115 (49.4)	0.023
Stage 2	37 (63.8)	21 (36.2)	
Stage 3	38 (63.3)	22 (36.7)	
Stage 4	17 (77.3)	5 (22.7)	
Median Total Testosterone (TT) (ng/ml)	294.5 [135–496]	482 [191–602]	< 0.001†
TT category			
≥300	103 (49.1)	107 (50.9)	0.001
<300	107 (65.6)	56 (34.4)	
Hypogonadism			
Yes	117 (64.3)	65 (35.7)	0.002
No	93 (48.7)	98 (51.3)	

*Fisher’s exact.

†Two-sample Wilcoxon rank-sum (Mann-Whitney) test.

### Factors associated with erectile dysfunction among study participants

In the univariate analysis the independent variables associated with ED were non-self-employment status (COR 2.8; 95% CI 1.6–5.1; p = 0.001)), WHO clinical stage 4 of HIV infection (COR 3.3; 95% CI 1.2–9.3; p = 0.023)), low testosterone level (< 300) (COR 2.0; 95% CI 1.3–3.0; p = 0.001) and hypogonadism (COR 1.9; 95% CI 1.3–2.9; p = 0.003). Following multivariate logistic regression analysis, newly diagnosed ART naïve MLWH who were non-self-employed (AOR 3.7; 95% CI 2.0–7.0; p< 0.001) were four times more likely to develop ED as compared to self-employed MLWH, newly diagnosed ART naïve MLWH in WHO clinical stage 4 for HIV infection (AOR 3.2; 95% CI 1.1–9.2; p = 0.032) were three times more likely to develop ED as compared to those in WHO clinical stage 1 for HIV infection and newly diagnosed ART naïve MLWH with low testosterone level (< 300) (AOR 1.9; 95% CI 1.2–3.0; p = 0.010) were two times more likely to develop ED as compared to those with normal and above testosterone level. Univariate analysis, CD4+ count (COR 0.6; 95% CI 0.3–1.2; p = 0.136), and hypogonadism (COR 1.9; 95% CI 1.3–2.9; p = 0.003) were not subjected to multivariate logistic regression analysis due to collinearity with WHO clinical stage and Testosterone (TT) respectively (**Table 4**).

**Table 4:** Factors associated with erectile dysfunction among newly diagnosed ART naive HIV-infected males (Univariate and multivariate logistic regression).

Characteristics	Erectile dysfunction		Univariate		Multivariate	
	Yes n (%)	No n (%)	COR (95% CI)	p-value	AOR (95% CI)	p-value
Age (Years)						
18-30	26 (50.0)	26 (50.0)	1.0		1.0	
31-45	118 (53.9)	101 (46.1)	1.2 (0.6–2.1)	0.614	1.1 (0.5–2.2	0.589
≥ 46	66 (64.7)	36 (35.3)	1.8 (0.9–3.6)	0.080	1.6 (0.7–3.6)	0.266
BMI (kg/m^2^)						
18.5–24.9	148 (56.5)	114 (43.5)	1.0		–	–
<18.5	34 (61.8)	21 (38.2)	1.2 (0.7–2.3)	0.468	–	–
25-29.9	25 (51.0)	24 (49.0)	0.8 (0.4–1.4)	0.480	–	–
≥ 30	1 (25.0)	3 (75.0)	0.3 (0.03–2.5)	0.242	–	–
Waist circumference (cm)						
81–93	90 (57.7)	66 (42.3)	1.0		–	–
<81	113 (54.8)	93 (45.2)	0.9 (0.6–1.4)	0.590	–	–
94–115	7 (63.6)	4 (36.4)	1.3 (0.4–4.6)	0.700	–	–
Marital status						
Single	40 (49.4)	41 (50.6)	1.0		1.0	
Married	146 (60.8)	94 (39.2)	1.6 (0.9–2.6)	0.072	1.4 (0.8–2.6)	0.253
Divorced/separated	24 (46.2)	28 (53.8)	0.9 (0.4–1.8)	0.716	0.6 (0.3–1.4)	0.243
Herbal medicine use						
No	131 (52.8)	117 (47.2)	1.0		1.0	
Yes	79 (63.2)	46 (36.8)	1.5 (0.9–2.4)	0.057	1.4 (0.9–2.40	0.156
Employment status						
Self-employed	144 (51.4)	136 (48.6)	1.0		1.0	
Non Self-Employed	51 (75.0)	17 (25.0)	2.8 (1.6–5.1)	0.001	3.7 (2.0–7.0)	**< 0.001**
Unemployed	15 (60.0)	10 (40.0)	1.4 (0.6–3.3)	0.413	1.6 (0.7–4.1)	0.284
CD4 count (cells/µl)						
>350	57 (55.3)	46 (44.7)	1.0		–	–
<200	53 (66.3)	27 (33.7)	0.7 (0.4–1.3)	0.232	–	–
200-350	48 (57.1)	36 (42.9)	0.6 (0.3–1.2)	0.136*	–	–
WHO clinical stage					–	–
Stage 1	118 (50.6)	115 (49.4)	1.0		1.0	
Stage 2	37 (63.8)	21 (36.2)	1.7 (0.9–3.1)	0.074	1.7 (0.9–3.2)	0.094
Stage 3	38 (63.3)	22 (36.7)	1.7 (0.9–3.0)	0.081	1.4 (0.7–2.6)	0.351
Stage 4	17 (77.3)	5 (22.7)	3.3 (1.2–9.3)	0.023	3.2 (1.1-9.2)	**0.032**
TT (ng/ml)						
≥300	103 (49.1)	107 (50.9)	1.0		1.0	
<300	107 (65.6)	56 (34.4)	2.0 (1.3–3.0)	0.001	1.9 (1.2–3.0)	**0.010**
Hypogonadism status						
No	93 (48.7)	98 (51.3)	1.0		–	–
Yes	117 (64.3)	65 (35.7)	1.9 (1.3–2.9)	0.003*	–	–

*These factors were not subjected to multivariate logistic regression analysis because of collinearity with WHO clinical stage and TT respectively. Bold p-value = significant.

COR, Crude Odds Ratio.

AOR, Adjusted Odds Ratio.

## Discussion

Erectile dysfunction (ED) is a global public health concern owing to its negative impact on the well-being of individuals, families, and communities. In this study, the prevalence of ED among ART naïve MLWH was found to be 56.3% (*95%* CI 52.1%–61.3%). This prevalence is notably higher than the prevalence rate (29.7%) among adult men in the general population (44) but lower than the prevalence rate (74.6%) reported among MLWH from Northern Tanzania (45). This finding is similar to the report by Falade et al. in Nigeria (46), Moreno-perez et al. in Spain (2) and Crum cianflone et al. in the US (28) which reported the prevalence of 57%, 53.4% and 61.2% respectively. On the other hand, our finding was higher than 37.8% and 21.6% rates reported by Adebimpe et al. in Ogbomoso, Southwest Nigeria (35) and Gomes and Brites in Brazil (3) respectively. The variation in these prevalence rates could be attributed to the differences in the research instruments used in assessing ED, characteristics of the study populations as well as sociocultural and geographical differences. Hypogonadism is one of the major cause of erectile dysfunction and report from previous studies have shown the existence of racial/ethnic variations in sex/gonadal hormone which may be due to genetic factors, socio-demographic factors, cultural factors, and/or environmental factors/dietary variations (5, 47–50). Also different tools may be defining ED differently and this might affect the results by either under reporting or over reporting ED cases leading to variations in the finding between different studies (51). For example in a current study, International Index of Erectile Function-5 (IIEF-5) questionnaire with five scored questions (ED: total score< 22). was used but the previous study by Gomes and Brites (3) in Brazil used IIEF-15 questionnaire which have 15 questions (ED: total score< 26) and Adebimpe et al. (35) in Nigeria used just a pre-tested semi structured questionnaire with unscored questions about erectile function.

Regarding categories of ED according to severity among participants, of the 210 subjects with ED in our study, 95 (45.2%) were mild ED, 84 (40.0%) were mild to moderate ED, 24 (11.4%) were moderate ED and 7 (3.3%) had severe ED. A closely similar pattern was reported by Mbwambo et al. in Northern Tanzania (45) and Falade et al. in North central Nigeria (46) where mild, mild-moderate, moderate and severe ED in Tanzania were found in 37.7%, 26.2%, 5.7% and 4.9% patients respectively and in 64.9%, 24%, 8.2% and 2.9% patients respectively in Nigeria. However, in Brazil, Gomes and Brites (3) found that only 13.7% of participants had a mild presentation of ED, while the remaining 86.3% of the study subjects presented with severe ED. Also, in a study done by Enoma et al. in Malaysia (52), ED was almost evenly distributed across all severity grades with severe (24.1%), moderate (19.1%), mild to moderate (20.9%), and mild (18.3%). This variation in the pattern of ED could be due to the use of different patient group, with distinct epidemiological, social and clinical characteristics as different patient group may differ in terms of various factors such as age, comorbid conditions (such as Diabetes mellitus and cardiovascular diseases), ART and other drug status, and life style habits of which have been also shown to have effect on gonadal hormone and erectile function (53–58). However, this requires further research for confirmation.

The risk factors for ED include vascular diseases, hypertension and obesity which are typically more common in the elderly population (59). Several studies have found a significant association between older age and the prevalence of ED (2, 16, 28, 35, 45), however, that was not detected in the current study. The study sample was relatively young (median age 40 [33–46] years) with the majority (58.7%) in the age range of 31 to 45 years. Furthermore, as most of the studies that report a significant association with age, involved HIV patients who were on ART, our study consisted of newly diagnosed ART naïve HIV males. Thus the previously reported relation of ED with young age and the association of ART with ED especially when the regimens contain protease inhibitors (21, 60, 61), could explain the observed discrepancy in this study. However, studies by Gomes and colleague (3) and Shindel et al. (30) among young HIV patients on ART (mean age 44 and 42 years, respectively) showed similar results but both studies included patients with comorbid conditions (Diabetes mellitus, Chronic pulmonary diseases, Hypertension) while Shindel et al. also included HIV-negative subjects in their study.

In our study, no significant association was found between marital status and ED. This finding corresponds to the reports by Gomez et al. (3) in Brazil, Mbwambo et al. (45) in Northern Tanzania, and Falade et al. (46) in Nigeria, and although is in contrast of those other studies which showed significant association between marital status and ED (58, 62–64). The finding from most previous studies with a significant association between marital status and ED indicated that single or divorced men were significantly associated with severe to moderate ED (62–64). Divorce/separation can involve significant changes in a person’s lifestyle, financial status, social connections and intimacy, which can negatively impact physical health including sexual health (62, 65). On the other hand, a study done in Thailand found that married men rate their sexual abilities better than single, divorced, separated, and widowed males (66). The variations in the findings could be due to differences in sociodemographic and clinical characteristics between study groups, as our study involved men living with HIV which is similar for most of previous studies reporting no significant associations and there were a low number of divorced/separated men among our study participants.

In this study, an association between herbal medicine use and ED was not observed. In a previous report, herbal medicine use was found to be significantly associated with hypogonadism in newly diagnosed ART naïve HIV-infected males (67). As hypogonadism is reported to be one of the factors causing ED (22), a positive association between herbal medicine use and ED was anticipated. The lack of such an association between herbal medicine use and ED in this study could be attributed to the small number of participants who reported to use herbal medicine. The higher rate of participants not using herbal medicine recorded in our study could be due to regional variations and the fact that patients with debilitating/systemic diseases were excluded from the study.

Findings of the present study show that employment status is significantly associated with ED and this is in support of the findings of previous studies (3, 58). This finding suggests that the development of ED is prone to be affected by employment status. Particularly, non-self-employed men were 3.7 times more likely to develop ED as compared to self-employed men. This finding could be attributed to financial instability (financial insecurity) leading to stress and anxiety related disorders causing ED.

A statistically significant association was also found between WHO clinical stage (that determines the clinical progression of the disease) and ED whereby MLWH in WHO clinical stage 4 for HIV infection were three times more likely to develop ED as compared to those in stage 1. HIV infection causes impaired immunity with an associated decrease in CD4+ count leading to comorbid conditions, hence poor health status. This observation may be explained by the link between poor health status and impaired gonadal function (6, 68). This finding is in support of the reports by Crum-Cianflone et al., which showed that a higher CD4 count was protective against ED but contrary to the findings by Mbwambo et al. which showed a lack of association between CD4 and ED. This difference could be attributed to the variation in the clinical characteristics of study participants, such as ethnicity, ART status and the prevalence of hypogonadism. Studies have shown racial/ethnic variation in infection acquisition and CD4 cell count as well as viral load and therefore these might reflect the WHO clinical stage for HIV infection in different populations (69–72).

Furthermore, this study shows that nearly half of the study participants had hypogonadism. Several studies have also demonstrated an association between HIV and low serum testosterone (28, 73, 74). Contrary to the previous studies by De Vincentis et al. (23) and Mbwambo et al. (45), the current study revealed a significant association between ED and low testosterone. This discrepancy could be due to variation in the number of participants with low testosterone levels. HIV can cause low testosterone, although currently, the relationship between HIV and testosterone is a controversial one, as other studies have found normal testosterone levels in MLWH (68, 75).

### Strengths and limitations

Limitations of this study include determination of ED only through self-report while it is recommended to establish ED diagnosis after a complete physical and psychological evaluation to determine vasculogenic or psychogenic factors (76, 77). However, ED in this study was determined using a validated IIF-5 questionnaire which is an internationally certified tool for assessing ED. The convenient sampling technique used in this study makes it prone to estimation bias and poor generalizability, however, this study was a multicenter study allowing generalization of the results. Measurement of gonadal hormones (TT, LH, FSH and estradiol) and socio-economic (employment) status allows assessment of both organic and psychological factors related to ED respectively. Also this study is limited by the use of TT to diagnose hypogonadism which can underestimate the prevalence of biochemical hypogonadism due to the possible rise in serum sex hormone binding globulin (SHBG) in HIV patients. Measurement of SHBG has been highly recommended, in addition to LH and TT in these patients (78, 79). Another limitation of this study is that, testosterone levels were determined using an immune-assay technique, whereas mass-spectroscopy is often considered “gold-standard” but is not commonly used because it is expensive and not widely available. However, the immuno-chemiluminescence assay used in the determination of the gonadal hormone values in this study is internationally certified and widely used in clinical practice to diagnose and guide treatment in patients with gonadal dysfunction.

In addition, this study is limited by failure to rule out factors that may also cause erectile dysfunction (by affecting neuron functions) such as hypertension, other sexually transmitted infections like syphilis, gonorrhea and chlamydia. Further in the current study, we did not include other factors such as alcohol use, cigarette smoking and education status of which other studies have shown there is an association between them and ED (2, 16, 28, 46).

This was the first study on ED in HIV patients in Northwestern Tanzania, and provides information on risk factors and magnitude of ED in our setting. Also this study being done among newly diagnosed ART-naïve HIV males allows observation of the impact of chronic inflammation of the virus itself rather than the role of ART which was not distinguished in most of the previous studies. Further studies are required to compare ED between men who are HIV positive and those who are HIV negative and follow-up studies to compare before and after initiation of ARTs.

## Conclusion

Slightly more than half of ART naïve MLWH were found to have ED with the majority presenting with a mild to mild-moderate form of ED. There was a significant association of ED with WHO clinical stage 4 for HIV infection, lower testosterone level, and being non self-employed. The results of this study emphasizes the need to include ED screening as part of the overall care of MLWH in CTC clinics as well as to strengthen the HIV screening program for early diagnosis and treatment to mitigate most of the observed risk factors for ED.

## Data Availability

The raw data supporting the conclusions of this article will be made available by the authors, without undue reservation.
